# Framing the Future with Bacteriophages in Agriculture

**DOI:** 10.3390/v10050218

**Published:** 2018-04-25

**Authors:** Antonet Svircev, Dwayne Roach, Alan Castle

**Affiliations:** 1 Agriculture and Agri-Food Canada, Vineland Station, ON L0R 2E0, Canada; 2 Department of Microbiology, Pasteur Institute, 75015 Paris, France; dwayne.roach@pasteur.fr; 3 Department of Biological Sciences, Brock University, St. Catharines, ON L2S 3A1, Canada; acastle@brocku.ca

**Keywords:** bacteriophage, phage therapy, sustainable agriculture, zoonosis, antibiotic resistance

## Abstract

The ability of agriculture to continually provide food to a growing world population is of crucial importance. Bacterial diseases of plants and animals have continually reduced production since the advent of crop cultivation and animal husbandry practices. Antibiotics have been used extensively to mitigate these losses. The rise of antimicrobial resistant (AMR) bacteria, however, together with consumers’ calls for antibiotic-free products, presents problems that threaten sustainable agriculture. Bacteriophages (phages) are proposed as bacterial population control alternatives to antibiotics. Their unique properties make them highly promising but challenging antimicrobials. The use of phages in agriculture also presents a number of unique challenges. This mini-review summarizes recent development and perspectives of phages used as antimicrobial agents in plant and animal agriculture at the farm level. The main pathogens and their adjoining phage therapies are discussed.

## 1. Introduction

The goal of sustainable agriculture is to implement practices that will attain healthy disease-free plants and animals, provide safe food for a growing global population, and minimize the impact of agricultural practices on the environment [1,2,3]. Conversely, agricultural practices are impacted by economic and disease pressures, consumer preferences, geographic location, weather conditions, and government regulations. Following the Second World War, antibiotics have been incorporated into animal husbandry [4,5] and for the control of plant pathogens [6,7,8,9,10]. Important strides in phage therapy were overshadowed by the widespread usage of antibiotics to treat diseases in humans, animal husbandry, and the control of bacterial plant pathogens. The overuse in medicine and animal husbandry has contributed to the rise of worldwide antimicrobial resistant (AMR) bacteria. Using *Erwinia amylovora* as an example, antimicrobial resistance is present in a number of geographic locations where antibiotics have been overused in apple and pear orchards [6,7,8,10]. The debate and scientific discussion on the impact and consequences of the presence of streptomycin resistant *E. amylovora* in orchards still continues in scientific literature [11].

Most antibiotics are non-specific, acting not only against the target pathogen, but also against other bacteria naturally present in the environment or plant and animal microflora. Drug-resistant infections result in millions of people being affected from drug-resistant bacteria each year, with an estimated 700,000 deaths worldwide each year, a number that could increase to 10 million by 2050 if the drug resistance trend continues [12]. Imprudent use of antimicrobials in agriculture may result in reduced efficacy of antibiotics due to facilitated emergence of antibiotic resistant human pathogens, increased human morbidity and mortality, increased healthcare costs, and increased potential for carriage and dissemination of pathogens. Together with consumers’ calls for antibiotic-free products, popularity of organic products and the removal of antibiotics for agricultural use in certain jurisdictions have led to the search for alternatives. Use of phages, which infect and destroy bacteria, could significantly reduce the environmental impact of antibiotic use in agriculture, while potentially increasing profitability by lowering crop loss or animal mortality in early stages of the breeding process.

## 2. Phages in Agriculture

“What is the impact of phages on agricultural environments where sustainable agriculture is being practiced?” becomes an important and intriguing question. Phages are inherently highly specific towards bacterial hosts. This characteristic has both negative and positive aspects in that it is beneficial in terms of avoiding negative effects on the host microbiota and a hindrance when it comes to detection and elimination of the target pathogen. This mini-review will focus primarily on the progress of phage-based biocontrol in food production systems covering the past 10 years. Phage-based laboratory studies that include phage isolation, host range determination, molecular characterization, genomic and proteomics analyses are well described in the recent review articles on plant and animal-associated phage therapy [13,14,15,16,17]. The development of phages as antimicrobial agents in animal and plant production systems follows a similar path in the initial discovery stage however the processes become divergent in the implementation processes. In the following sections we discuss the progress made in the use of phages in plant and animal farming, focusing on the challenges and success stories reported in scientific literature.

## 3. Bacteriophages in Food Animal Production

By volume, the vast majority of antibiotics consumed worldwide are for veterinary purposes, predominantly in intensive and large-scale animal production systems, such as dairy, livestock, poultry, and aquaculture [18,19]. Animal husbandry practices widely use antibiotics therapeutically to treat infectious diseases, as well as non-therapeutically to prevent the spread of disease (prophylaxis) and to promote growth. Controversy, however, surrounds the widespread use of antibiotics for animal production, as their overuse and possible misuse is driving antibiotic microbial resistance. For instance, the practice of prolonged exposure to sub-therapeutic antibiotic doses, the context in which prophylactic and growth-promoting antibiotics are administered, exerts an inestimable amount of selective pressure toward the emergence of AMR [20,21]. Furthermore, AMR bacteria and AMR genes of animal origin can then be transmitted to humans through environmental contamination, food distribution, or direct contact with farm animals [22,23,24]. Intensive animal production systems necessitate antibiotics to keep animals healthy and maintain productivity, and with rising incomes in transitioning countries expected to boost antibiotic consumption by 67% by 2030 [25], this presents a major health risk to humans and animals.

The World Health Organization, the European Commission, the Centers for Disease Control and Prevention, and Health Canada, to name a few, all support immediate antimicrobial stewardship in animal food production, aimed primarily at reducing or eliminating the nontherapeutic use of medically important antibiotics. Eliminating prophylactic antimicrobials outright may not be feasible in intensive animal production systems due to increasing worldwide demand for protein, the potential compromise in animal welfare and health, and in human health and food safety. Phages instead of antibiotics are a promising option in food animal production to maintain animal health and limit the transfer of AMR and zoonotic pathogens that may be harmful to consumers. This section will focus only on application of phages as alternatives to antibiotic growth promoters, prophylaxis, and zoonotic pathogen animal decolonization at the farm level.

### 3.1. Phages as Growth Promoters

Antibiotics in subtherapeutic doses have played important roles in the promotion of growth, enhancement of feed efficiency and improvement of the quality of animal products [20]. To combat the increased rate of mortality and morbidity due to reduction of in-feed antibiotics, phages have been proposed as the replacement, particularly in the early stages when vaccination is not possible and the maintenance of the bacterial ecosystem is crucial [26]. The studies reviewed in this subsection highlight the addition of phages in feed rather than for clinical treatment. The distinction between growth promotion and prevention or treatment of diseases is subtle and further work is needed to see if phages do offer growth promotion effects other than simply reducing disease incidence.

*Clostridium perfringens* is a major problem for the poultry industry, resulting in both clinical and subclinical infections. A cocktail of five phages could effectively control necrotic enteritis in chicken broilers and thus improve feed conversion ratios and weight gain [27]. This efficacy was independent of whether the phages were administered in feed or in drinking water. Dietary supplementation with phages has also been shown to improve on growth performance in pigs [28]. Feed supplemented with a commercial phage product, which contained a mixture of phages targeting several pathogens, including *Salmonella* spp., *Escherichia coli*, *Staphylococcus aureus*, and *C. perfringens*, improved different aspects of grower pig’s performance, such as average daily feed intake [28]. It was determined that barrow gut health improved with a higher abundance of commensal bacterial and lower pathogen load in pig faeces. However, the success of phages against pathogenic bacteria could be related to the method of its addition. Administering phages in drinking water may be disease and/or pathogen dependent. Huff et al. [29] found that phage administered in drinking water could not cure experimental *E. coli* respiratory infections in broilers. Phages were only effective in reducing respiratory bacterial load when they were administered via direct intratracheal administration [30].

For dairy herds, mastitis is the most important disease worldwide [31]. *S. aureus*, one of the etiological agents for mastitis, which has a propensity to recur chronically, causes a potentially fatal inflammatory response in gland tissues. In an experimental model, lactating mice intramammarily infected with a clinical bovine *S. aureus* strain showed significant improvement in mammary gland pathology and a 4-log reduction in bacterial load after phage treatment [32]. However, compared to the antibiotic cefalonium, the phage treatment was far less effective. Gill et al. [33] also found that multiday high-titre intramammary infusions of phage K did not lead to a reduction in *S. aureus* load in the utter of lactating cows with pre-existing subclinical mastitis [34]. In this latter study, the adsorption of milk whey proteins to the *S. aureus* cell surface inhibited phage infection in vitro, suggesting this was the cause for treatment failure [33]. It should be noted that antibiotic treatment success is also highly variable with mastitis cure rates as low as 4% [35].

Phages have the potential to be a viable and eco-friendly alternative to antibiotics in aquaculture. Aquaculture is the fastest growing food production sector, providing over fifty percent of the world’s supply of fish and seafood. Antibiotics in feed are commonly used as prophylactics to decrease the corresponding heavy economic losses due to bacterial diseases worldwide. Vibriosis is one of the most prevalent diseases of marine and estuarine fish in both natural and commercial production [36,37,38]. *Vibrio anguillarum* is the etiologic agent of vibriosis, a fatal haemorrhagic septicaemia that affects more than 50 fresh- and salt-water fish species including several important food species, such as the Atlantic salmon, rainbow trout, turbot, sea bass, and sea bream [36]. A single phage treatment protected 100% of Atlantic salmon against experimentally induced *V. anguillarum* infection [39]. Vibriosis also causes high mortality rates in fish larvae. Phages administered in culture water of zebrafish larvae experimentally infected with *V. anguillarum* significantly lowered larvae mortality [38]. Likewise, phages added to culture water of shrimp larvae improved survival after experimental infection with *Vibrio harveyi* [40]. Thus, directly supplying phages to the culture water could be an effective and economical approach toward reducing the negative impact of vibriosis in aquaculture, in particular when vaccines are not an option to protect larvae.

Other experimental aquaculture models have also shown promising phage efficacy. For instance, phages in-feed has been shown to protect against water-borne *Pseudomonas plecoglossicida* infection, the etiological agent of bacterial haemorrhagic ascites in freshwater fish, including ayu, pejerrey, rainbow trout, and large yellow croaker [41]. In a field trial, phage-impregnated feed was added to the fishpond where *P. plecoglossicida* was naturally present and daily ayu mortality of fish decreased by 30% after multiple weeks of prophylaxis. Moreover, neither phage-resistant bacteria nor phage-neutralizing antibodies were detected in infected or cured fish [42].

### 3.2. Phages that Combat Zoonotic Pathogens

Phages offer a non-antibiotic method to improve food safety as a preharvest intervention to reduce zoonotic pathogens from the food supply. For instance, contaminated poultry, pork, beef, and fish have led to food poisoning and food-related disease. Often, food-borne pathogen contamination of meat products occurs during processing when carcasses are exposed to infected animal faeces. Campylobacteriosis caused by *Campylobacter jejuni*, is the most frequent food-borne human enteritis in developed countries, the major source being tainted poultry meat. Loc Carrillo et al. [43] showed that an antacid solution containing phages given orally could effectively decolonize the gut of birds experimentally colonized with *C. jejuni*. Under commercial conditions, however, phage decontamination success was highly variable. When a phage cocktail was added to the drinking water at three commercial farms with broilers confirmed to be colonized with *Campylobacter* spp., only one farm experienced a reduction in bacterial load (<50 CFU/g) in faecal samples [44]. For the other two farms, no significant reduction occurred for undetermined reasons.

Salmonellosis is another common cause of gastroenteritis in humans. Pigs can become colonized with *Salmonella* spp. from contaminated trailers and holding pens, resulting in increased pathogen shedding just prior to processing. Wall et al. [19] showed that administration of a phage cocktail at the time of experimental inoculation with *Salmonella enterica* serovar Typhimurium reduced bacterial load to almost undetectable limits in the tonsils, ileum, and cecum of infected small pigs. A phage cocktail significantly reduced cecal and ileal *Salmonella* concentrations by up to 95% after being in a highly contaminated holding pen. *S. enteritidis* is also a prevalent foodborne pathogen, its main reservoir being the eggshell. Use of a mixture of three different *Salmonella*-specific phages to reduce *S. enteritidis* colonization in the ceca of laying hens resulted in a significant decrease in bacterial prevalence of incidence of up to 80% [45].

*E. coli* is typically a commensal member of human and animal microbiota. However, certain strains can cause a variety of human diseases, including urinary tract infections, haemorrhagic colitis, appendicitis and septicaemia. The most notorious zoonotic strains are those referred to as Vero-Toxigenic *E. coli* (VTEC). The most common member of this group is strain O157:H7 and the natural reservoir is the cattle gut. A cocktail of phages isolated from cattle faeces was able to reduce O157:H7 populations in the gut of experimentally inoculated sheep, with a 1:1 ratio of phage to bacteria found to be more effective than higher phage ratios [46]. Upon necropsy, *E. coli* populations were found to be reduced in both the cecum and colon, while ruminal load was not significantly changed, likely due to a relatively low starting population [46].

## 4. Bacteriophages in Crop Production

The discovery research on phages and plant pathogens took place nine years following the highly disputed discovery of phages by Frederic Twort in 1915 and Felix D’Herelle in 1917 [47]. The first experimental evidence that phages may be associated with plant pathogenic bacteria occurred when it was demonstrated that a filtrate obtained from decomposing cabbage was able to inhibit cabbage-rot caused by *Xanthomonas campestris* pv. *campestris* [48]. The following year, Kotila and Coons [49] demonstrated that the exposure of *Pectobacterium atrosepticum* to phages prevented the development of soft rot in potatoes. The first recorded field trial occurred in 1935, when Stewart’s wilt disease of corn, caused by *Pantoea stewartii*, was reduced by pre-treatment of seeds by phages [50].

In a 2012 survey, bacterial pathologists that read the Journal Molecular Plant Pathology were asked to list three important plant pathogens [51]. The top 10 plant pathogens listed in descending order were *Pseudomonas syringae, Ralstonia solanacearum*, *Agrobacterium tumefaciens*, *Xanthomonas* spp., *Erwinia amylovora*, *Xylella fastidiosa*, *Dickeya* spp., and *Pectobacterium* spp. Lack of chemical control options and development of antibiotic resistance in many plant pathogens combined with consumers’ preference for organic and antibiotic free products has led to a phage therapy renaissance in agriculture.

### 4.1. Soft Rot, Bacterial Wilt, and Blight

*Dickeya solani* and *Pectobacterium* spp. are pathogens associated with potato tuber soft rot in storage and blackleg disease in the field [52,53]. Adriaenssens [52] used two *Dickeya* sp. *Myoviridae* phages as biological control agents for the control of soft rot/blackleg in potato. Potato tubers were vacuum infiltrated with the pathogen and the phages were sprayed (nebulised) at MOI of 10 and/or 100 over the infested tubers. Treated tubers were planted in the field and the disease progression was monitored through the growing season. There was no significant difference between the treated control, untreated controls, and the phage/bacteria treatments. To study the ability of the phage mixtures to control multiple pathogen species associated with bacterial soft rot, two broad host range phages [53] and 9 phage mixtures [14] were tested on potato slices but not in the field. Czajkowski [14] provides in a recent review a detailed summary on the advances in research on the phages of the soft rot bacteria.

Pre-treatment with a *Podoviridae* phage PE204 under growth chamber conditions did not achieve control of *R. solanacearum*, the cause of bacterial wilt of tomato [54]. Single phage and/or cocktails composed of commercial phage mixtures were applied as a soil drench with an attenuated *Xanthomonas perforans* isolate. Phage populations were followed in the treatments in the root zone and the inside of plants. Partial translocation of phages occurred into the lower portions of the tomato plant and greenhouse and field trials demonstrated that in the presence of *X. perforans* mutant phage populations increased on leaf surfaces and in the soil [55].

*Pseudomonas syringae* pathovars are responsible for a large number of plant diseases in agriculture [51,56]. The recent serious global outbreak of *P. syringae* pv. *actinidae* in kiwifruit production and the lack of control options has re-focused research onto phages [57,58]. To date, this research has focused on phage characterization by host range and genomic studies. Two parallel field trials in three locations were conducted for the control of *P. syringae* pv. *porri*, bacterial blight of leek [59]. The treatments involved a 6-phage cocktail and plants that were either pre-treated with phage and then infected by the pathogen or treated with pathogen followed by the phage cocktail at 10^9^ pfu/ml. Statistically significant difference between treatment and control were not obtained and the results were highly variable between the locations.

### 4.2. Citrus Bacterial Canker and Spot

Balogh (2008) studied phage-mediated control of *Xanthomonas axonopodis* pv. *citri*, Asiatic citrus canker (ACC) and *X. axonopodis* pv. *citrumelo*, citrus bacterial spot (CBS). Treatments without skim milk, used to stabilise the phage, additive significantly reduced ACC disease severity. In nursery trials, the ability of phage mixtures, copper-mancozeb, and the combination of phage-copper-mancozeb to control CBS and ACC were tested. Phages reduced ACC disease significantly but were not as effective as the copper-macozeb treatment alone. The phage-copper-mancozeb combined treatment failed. Similar results were seen for CBS, where phage control was significantly different from the control in Valencia oranges but not in grapefruit under low disease pressures [60]. Ibrahim et al. [61] obtained successful control of Asiatic citrus canker in greenhouse and field trials, by combing a compound which induced the plants systemic acquired resistance and phage mixtures formulated in skim milk and sugar.

### 4.3. Pierce’s Disease of Grape

*Xylella fastidiosa* is a pathogen of a number of plants but it has the greatest economic impact in grapes. Disease control options are limited and challenging since the pathogen is limited to the xylem of the grape [62]. Recently, two lytic phages of *X. fastidiosa* subsp. *fastidiosa* have been isolated and fully characterised [63]. Phage cocktails in grape using therapeutic and prophylactic treatments were able to significantly control the pathogen and symptom development in greenhouse trials.

### 4.4. Fire Blight in Apples and Pears

The causal organism of fire blight, *Ewinia amylovora*, is a major pathogen in commercially grown apples and pears. The pathogen can exist in asymptomatic tissue or as an epiphyte in the orchard ecosystem [9]. All commercially desirable apple and pear cultivars are moderately to highly susceptible to this pathogen and resistant germplasm is not available. In Canada and the US, streptomycin and kasugamycin are applied during open bloom to obtain control of the fire blight pathogen [6,11]. In growing regions where streptomycin resistance is present and/or organic fruit is grown, the use of antibiotics is prohibited and alternative control strategies for integrated pest management practices are urgently needed.

Phages combined with *Pantoea agglomerans,* non-pathogenic host, belonging to the *Myoviridae* and *Podoviridae* have been tested under greenhouse [64] and field conditions [16,65] for their ability to control the pathogen during open bloom. The highly variable seasonal variation in biological control is not uncommon and it serves as one of the biggest challenges to the commercial development of phages in agriculture.

### 4.5. Impact of Host Exopolysaccharides and Phage Family on Efficacy

*E. amylovora* pathogenicity is largely determined by the presence of amylovoran, a capsular exopolysaccharide (EPS), while virulence is associated with levan, a secondary component of the bacterial capsule [66]. Roach et al. [67] showed that the structure of the host cell surface plays a very important role in phage pathogenesis. Isolates of *E. amylovora* characterised as producing relatively large amounts of EPS, were called high EPS producers (HEPs) and low producers were labelled low EPS producers (LEPs). Phages in the *Myoviridae* grew better on LEPs than HEPs. In contrast, most but not all *Podoviridae* phages exhibited improved replication on HEPs hosts as measured by efficiency of plating. Deletion of genes required for the production of amylovoran and levan provided further insight into the function of the cell surface in phage growth. Deletion of the *rcs*B gene, which prevented synthesis of the EPS component amylovoran, resulted in almost complete resistance to most podoviruses tested. The effect of this deletion on myoviruses was variable with one phage showing a reduction in the efficiency of plating (EOP) and two others showing an increase, suggesting that amylovoran is likely not a significant contributor to phage pathogenesis for these phages. In contrast, deletion of the levansucrase gene, *lsc*, had little impact on the pathogenesis of podoviruses but resulted in a reduction of EOP by one to two orders of magnitude for myoviruses. These observations have three important implications on the impact of the use of these phages in a program to control *E. amylovora*. First, phages in the *Podoviridae* will likely have little to no impact on other bacterial epiphytes in the orchard because amylovoran synthesis is limited to *E. amylovora.* This prediction has been validated by tests on *P. agglomerans*. This epiphytic species does not produce amylovoran and the majority of the isolates do not support the growth of *E. amylovora* podoviruses). Second, one of the likely mechanisms by which *E. amylovora* could become resistant would be through a mutation that prevents amylovoran production. This would result in an avirulent bacterium that would greatly reduce the chance of survival. Thus, podoviruses should be included in any biocontrol formulation with the goal of reducing fire blight. Third, myoviruses should also be included in the formulation to increase the probability of inhibiting growth of all *E. amylovora* strains, including the LEPs. 

## 5. Potential Problems with Phages as Biocontrol Agents

The development of phage resistance in the bacterial host is a major concern in phage therapy. Just as bacteria may become resistant to antibiotics they may also become resistant to phages by a variety of mechanisms. These include modification of the phage surface receptors on the bacterial cell such as conversion to mucoidy [68], integration of the phage genome into the bacterial chromosome [57,69], restriction/modification systems [70], CRISPR/Cas systems [71,72], BREX [73], DISARM [74], and up to 9 new defense systems [75]. To prevent the development of bacterial resistance to phages the standard adopted practice has been to use a mixtures or cocktails that may contain combinations of phages with host ranges that are narrow, wide and/or composed of host range mutants [27,53,59,60,76]. One intriguing possible outcome of the use of a phage mixture is bacteria that are resistant to a particular phage can still be lysed by that phage through the acquisition of phage receptors from lysed sensitive cells. This effect has been observed during infection of *Bacillus subtilis* with phage SPP1 [77]. It will be important to investigate if the transfer of receptors is a phenomenon that extends well beyond this one example.

Another potential hurdle with the use of phages as biological agents is the production of lysogens or pseudolysogens. Persistence of the phage genome in the host cell would provide superinfection immunity that would negate the efficacy of the biological and possibly impart novel characteristics to the target bacterium. For example, ΦRSS1, a phage that exists in a persistent infective state in *R. solanacearum* increases virulence of the bacterial host on tomato [78]. Although this risk clearly exists, the scope of the problem remains poorly understood. Roach et al. [69] examined the prevalence of lysogens of myoviruses and podoviruses in 161 isolates of *E. amylovora* and 82 of *P. agglomerans*. None was detected. Use of phages to recover bacteriophage insensitive mutants (BIMs), however, showed that lysogeny was possible with the recovery of one stable lysogen. In addition, PCR analysis indicated that phage DNAs could be detected in subcultures of numerous BIMs for up to a year after selection although the association of phage and host was unstable. The authors concluded that though lysogeny could occur, it was likely to be selected against in the resource rich environment of the apple or pear blossom. As such, the risks associated with lysogeny were low. Nonetheless, this possibility should be considered for any application of phages for biocontrol.

A third potential hurdle is that phages could serve as vectors for mobile genetic elements, including antibiotic resistance genes [79,80]. Colavecchio et al. [81] recently reviewed the literature on the role of phages in the spread of AMR genes amongst members of the *Enterobacteriaceae*. These genes could certainly be transferred horizontally by transducing phage particles. The contribution of transduction to AMR spread, however, may be low as compared to conjugation or transformation. This issue is currently unresolved and deserves further attention.

Many bacterial pathogens form biofilms, which in turn impact phage therapy. In *E. amylovora*, amylovoran and levan contribute to the formation of a biofilm [82], yet phage efficacy bioassays continue to be carried out in liquid cultures. Today, models of phage–host interactions should take into consideration that biofilms form a spatial environment where resources are concentrated and bacterial materials and debris build up as cell numbers increase [83]. All these factors will influence the ability of the phage to adsorb and kill the bacterial host. Laboratory studies that use liquid cultures to study phage-host interactions are poor indicators of phage efficacy under greenhouse and/or field conditions. In a recent publication, Abedon [84] provides an excellent treatise on how phage therapy can be improved by incorporating important standards such as the Poisson distribution curve when reporting on the infection of host cells by phage and the avoidance of the commonly used MOIs to report phage dosages.

## 6. Discussion

Present-day research indicates that phages have the potential as an alternative control mechanism for eliminating pathogens posing a threat to animals and plants (Table 1), particularly with the increased risk of AMR and regulatory restrictions on the use of antibiotics in agriculture. Phages developed for the control of plant and animal pathogenic, zoonotic, and problematic bacteria exploit the multiple and complex host–microbe interactions to significantly reduce disease, reduce economic losses, and minimize the effect on the environment and on non-target microorganisms. In animal production, the focus of using phages as antimicrobial agents has been on controlling human and zoonotic pathogens.

In plant agriculture, control with phages has been difficult to implement due to a number of challenges. These include development of formulations to effectively treat hectares of plants grown in monoculture and/or in greenhouse conditions, assessing susceptible hosts including both bacterial pathogen and plant interactions as well as phage–bacterium matches, persistent pathogen presence, transmission of the pathogen by wind, rain, and insects, modern day farming practices that rely on chemical pesticides that may be deleterious to the phage, and unpredictable weather patterns within and between growing seasons. Timing of the biocontrol delivery is crucially important. Therapeutic treatments may involve phage application to reduce a pre-existing pathogen population or an application timed to the expected arrival of the pathogen [52,55,59,64,85]. For prophylactic treatment, phages are introduced prior to the anticipated appearance of the pathogen [59,85]. Efficacy of both options should be evaluated as part of a biocontrol development program. Aerial phage applications require formulations that will ensure the survival of the phage in the environment [60,61,76,86]. The alternative application methodology is to utilize a living bacterial cell delivery system that ensures survival and continued replication of the phages prior to the arrival of the pathogen [16,65]. For example, live cells of an attenuated bacterial strain of *Xanthomonas perforans* were used to improve the persistence of the phage populations in and on the soil [55].

In animal production, much of the focus of using phages as antimicrobial agents has been on controlling bacterial infection. The benefits of antibiotics in animal feed have added benefits in production. For instance, Thomke and Elwinger [87] hypothesize that cytokines released during the immune response may also stimulate the release of catabolic hormones, which reduce muscle mass. In addition, there is evidence that antibiotics suppress microbial fermentation in the gastrointestinal tract improving feed conversion by up to 6% (Jensen, 1998). Recent studies showed that a sub-therapeutic antibiotic correlates with the decreased activity of bile salt hydrolase, an intestinal bacteria-produced enzyme that exerts negative impact on host fat digestion and utilization [88]. Regardless of the mechanism of action, the use of animal growth promoters can improve daily growth rates between 1% and 10% resulting in meat of better quality with less fat and increased protein content. It will be important to explore whether phages provide similar growth enhancing effects beyond the benefits of controlling infectious diseases. Phages can also be used in post-slaughter or later processing systems as decontaminants, including the FDA approved commercial products ListShield™ (Intralytix, Baltimore, MD, USA) and PhageGuard L™ (formerly Listex™) (Micreos Food Safety B.V., Wageningen, Netherlands) as food additives for prevention of meat contamination with *Listeria monocytogenes* [89]. EcoShield™ (Intralytixs) for *E. coli* and SalmoFresh™ (Intralytix) for *Salmonella* spp. are also FDA approved to decontaminate ready-to-eat meat and poultry, fish and seafood, and dairy products.

Plant and animal phage development systems in food agriculture have their own distinct and specialised processes, protocols, and challenges. Regardless of the agricultural application, the process itself should be better defined, organized, and laid out. The science innovation chain for the development of biologicals or biopesticides was developed by Boyetchko [90]. This model defines and designates specific steps and processes that workers should address in the developed of phage biologicals (synonym in agriculture biopesticide). The project deliverables, arranged in continuous and ascending order, include acquisition of scientific knowledge, greenhouse/field/animal efficacy trials, fermentation/formulation, defining of markets, license agreements, large scale field test, manufacturing/process engineering, production of phage product, and product sales/client adoption. Concurrent with the deliverables and in the same ascending order, a series of stages and/or gates include discovery and selection of phages, proof of concept that the therapy works, technology development, market identification, technology transfer, commercial scale up, registration/regulatory processes, and technology adaptation by end users. This type of a model takes into consideration work beyond the laboratory and basic science and provides basic guidelines to the processes and decision points that need to be addressed during the development of a phage biological that can be successfully used in agriculture.

## Figures and Tables

**Table 1:** Experimental studies using bacteriophages to control bacterial pathogens.

Target Species	Disease/Issue	Animal/Plant	Study
*Clostridium perfringens*	necrotic enteritis	poultry	[27]
*C. perfringens*, *E. coli*, *S. aureus*	weight gain	swine	[28]
*Escherichia coli*	respiratory infection	poultry	[29,30]
*Staphylococcus aureus*	mastitis	bovine	[32,33]
*Vibrio anguillarum*	vibriosis	fish	[38,39]
*Vibrio harveyi*	vibriosis	shrimp	[40]
*Pseudomonas plecoglossicida*	haemorrhagic ascites	fish	[41,42]
*Campylobacter jejuni*	zoonotic	poultry	[43,44]
*Salmonella enterica* serovar Typhimurium	zoonotic	swine	[19]
*Salmonella enteriditis*	zoonotic	poultry	[45]
*Escherichia coli*	colitis	sheep	[46]
*Xanthomonas campestris* pv. *campestris*	cabbage rot	cabbage	[48]
*Pectobacterium atrosepticum*	soft rot	potato	[49,52]
*Pantoea stewartii*	Stewart’s wilt	corn	[50]
*Dickeya solani, Pectobacterium* spp.	soft rot/blackleg	potato	[52,53]
*Ralstonia solanacearum*	bacterial wilt	tomato	[54]
*Pseudomonas syringae* pv. *actinidae*	canker	kiwifruit	[57,58]
*Pseudomonas syringae* pv. *porri*	bacterial blight	leak	[59]
*Xanthomonas axonopodis* pv. *citrumelo*	bacterial spot	citrus	[60]
*Xanthomonas axonopodis* pv. *citri*	canker	citrus	[61]
*Xylella fastidiosa*	Pierce’s disease	grape	[63]
*Erwinia amylovora*	fire blight	apple/pear	[16,64,65]
